# ForageFeeder: A low-cost open source feeder for randomly distributing food

**DOI:** 10.1016/j.ohx.2023.e00405

**Published:** 2023-03-01

**Authors:** Nima Jadali, Margaret J. Zhang, Andrew K. Schulz, Josh Meyerchick, David L. Hu

**Affiliations:** a College of Computing, Georgia Institute of Technology, Atlanta, GA 30332, USA; b George W. Woodruff School of Mechanical Engineering, Georgia Institute of Technology, Atlanta, GA 30332, USA; c School of Biological Sciences, Georgia Institute of Technology, Atlanta, GA 30332, USA; d Zoo Atlanta, Atlanta, GA 30315, USA; e Max Planck Institute for Intelligent Systems, Stuttgart, Germany

**Keywords:** Conservation technology, Forage feeding, Behavioral biology, Deer feeding

## Abstract

Automated feeders have long fed mice, livestock, and poultry, but are incapable of feeding zoo animals such as gorillas. In captivity, gorillas eat cut vegetables and fruits in pieces too large to be dispensed by automated feeders. Consequently, captive gorillas are fed manually at set times and locations, keeping them from the exercise and enrichment that accompanies natural foraging. We designed and built ForageFeeder, an automated gorilla feeder that spreads food at random intervals throughout the day. ForageFeeder is an open source and easy to manufacture and modify device, making the feeder more accessible for zoos. The design presented here reduces manual labor for zoo staff and may be a useful tool for studies of animal ethology.

**Specifications table**

*Please replace the italicizied instructions in the right column of the table with the relevant information about your hardware.*

**Table:** 

**Hardware name**	**ForageFeeder**
**Subject area**	• Neuroscience • Biological Sciences (e.g. Microbiology and Biochemistry) • Environmental, Planetary and Agricultural Sciences • Educational Tools and Open Source Alternatives to Existing Infrastructure
**Hardware type**	• Biological sample handling and preparation • Field measurements and sensors • Conservation Technology
**Closest commercial analog**	The closest current commercial analogs are automated deer feeders that do not work for a range of the desired food items such as cut vegetables and fruits. They are also more expensive (cost around $1000-$2000) and do not easily accommodate customization.
**Open source license**	GNU GPLv3 License
**Cost of hardware**	Approximate Cost - $400**(Bill of materials).**
**Source file repository**	DOI repsitory can be found with all files here: http://doi.org/10.17605/OSF.IO/VAX5F

## Hardware in context

There are currently over 350 gorillas in AZA zoos captivity in North America [1]. Due to destruction of tropical rain forests in Uganda, Rwanda, and Congo, only 1000 mountain gorillas remain in their wild habitats. Even the most numerous and widespread of the gorilla subspecies, the western lowland gorilla, is listed as critically endangered due to habitat loss [1]. Given the quickly diminishing habitats of gorillas, there are many reasons to maintain some gorilla populations in captivity: for their own safety, for behavioral scientific research on their health and well-being, and for educating the public and encouraging conservation. The objective of this work is to design and build a feeder, which we call ForageFeeder, to elicit foraging behaviors in captive gorillas.

The idea for this project came from three zoo staff, one of them a coauthor, who have over 30 years of combined experience caring for primates at Zoo Atlanta. The Zoo Atlanta staff advised on the constraints, use, and maintenance of the proposed feeder. The remaining coauthors are engineers and computer scientists who have expertise in design and building. We believe that future devices for conservation tools may be found through collaboration with such interdisciplinary teams [2].

**Fig. 1:**
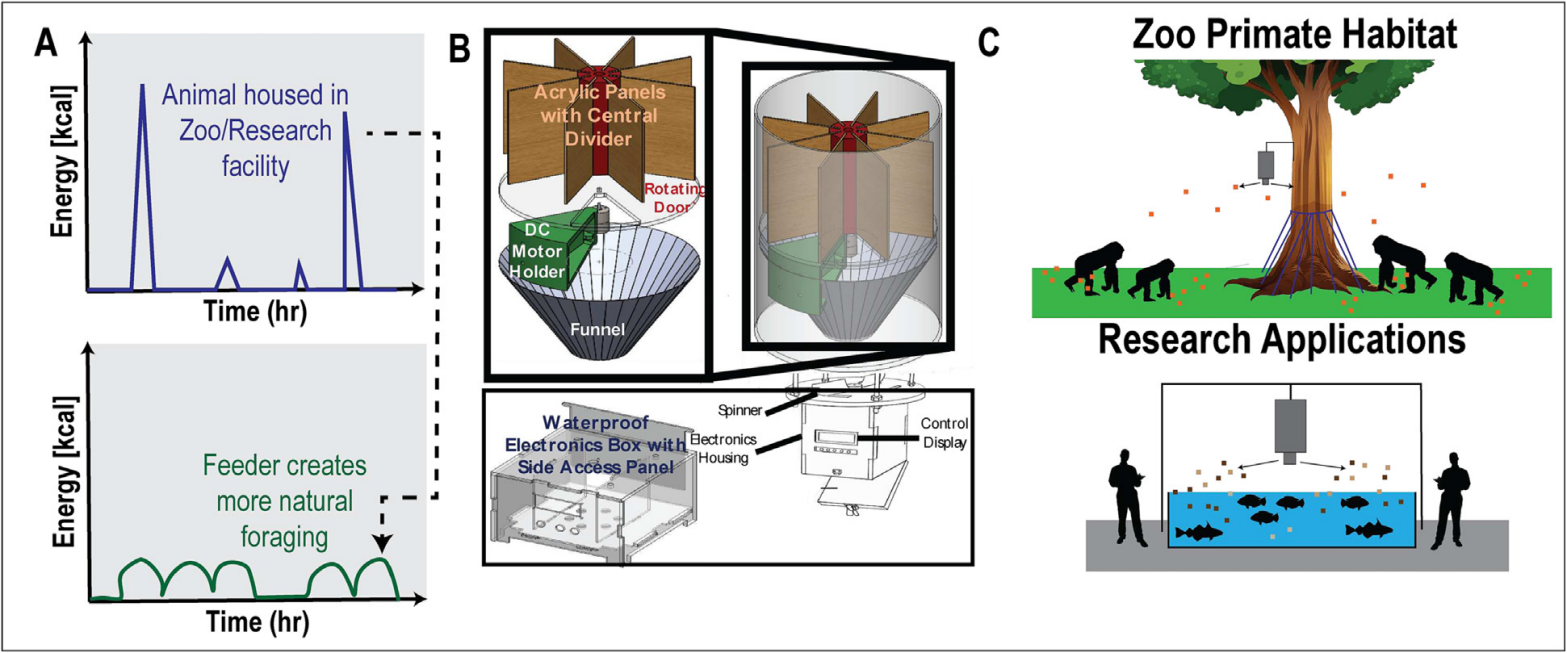
A) Hypothesized time course of energy intake for gorillas fed manually (top) and the forage feeder.  Energy graphs adopted from [3]. B) Proposed automated feeder. C) Envisioned applications of the feeder.

Due to limitations in staff time at most zoos, many species in captivity are manually fed during set mealtimes, similar to a human schedule. Each meal is associated with a spike in energy intake, which also correlates with spikes in blood sugar and body movement, as shown in Fig. 1A. This pattern is distinct from natural foraging in which gorillas eat and move continuously during daylight hours. The human-centered feeding schedule disrupts them from a natural feeding behavior. Influenced by factors such as physical ability, social hierarchy, and food availability, feeding behavior in wild primates are an important part of their daily lives [3]. Thus, restoring their natural feeding can provide a source of enrichment and more natural social interactions than human-based feeding.

Automated feeders are used primarily in farming and hunting sectors [4]. Because of these sectors’ target audiences and use cases, their feeders are designed to spread solid, dry food pellets which flow freely and are less prone to getting stuck. However, gorillas are fed fresh, damp deformable foods such as diced sweet potatoes or carrots, which are not compatible with commercial feeders.

While ground-based feeders can cost only one hundred dollars, other feeder solutions are often too expensive, forcing zookeepers to spend thousands of dollars on ill-suited feeders [5]. Additionally, devices used in zoological organizations are often over-engineered and not user-friendly [6]. Our goal is to make a foraging-behavior inducing, open-source feeder that can be easily built by zookeepers, scientists, and conservationists around the world with parts that are affordable, accessible, and easy to maintain.

Automated feeders designed by Oh et al.  release small amounts of food in an enclosed area [7]. To build a feeder that spreads feed over a larger area, we modified the design of Oh et al. and combined it with the food spreading capabilities of a typical deer feeder [8]. While designed for gorillas, we believe the device could be applied to any animal that forages over a large area, including elephants [9], fruit bats [10], and mule deer [11]. The ability to program the device to feed at different intervals may be useful in studies of animal behavior, growth, and nutrition. For example, Ali and Wooton studied the growth of stickleback fish due to feeding at both regular and random intervals [12].

We designed our feeder to be built frugally and open source so that it can be used by non-profit organizations such as zoos. Recent frugal devices have been proposed such as foldscope and paperfuge [13], [14], but frugal science has yet to gain momentum in zoological organizations [15]. Frugal science and open source designs are starting to make waves in the conservation biology community through the field of Conservation Technology (CT), which encapsulates technological developments for wildlife and environmental conservation [16], [17]. Some of the major goals of CT are to improve outdated equipment, increase accessibility to tools, and use modern technology to address conservation problems in entirely new ways [18], [2]. CT can be used as a less invasive method for understanding wildlife through various methods like data collection, monitoring, and automatic wildlife distribution [19]. Previous conservation technology works include the AudioMoth, an acoustic monitoring device, inexpensive camera traps, but there are few examples of animal feeders [18].

ForageFeeder has a total cost of approximately $400 with a majority of the cost associated with the instrumentation and motor control. We designed it to be impenetrable to squirrels, raccoons, and foxes, but this ability still remains untested. Non-electrical components were laser cut, 3D -printed, or inexpensively store bought. Electrical components were built from open-source parts. The LCD screen and buttons were optional, but helped to create a user-interface that could be more easily operated.

## Hardware description

In this section, we present the building process for the feeder. Supplemental Video 1 shows a time-lapse video of constructing the entire device from scratch, which takes approximately 2 h. Most components are designed to fit together easily and have minimum points of attachment and need for adhesive. This reduces the amount of pieces in the design and overall complexity of construction.

The design goals for the ForageFeeder include:

• Total cost of approximately $400
• Assembly in under 3 h
• Accessible maintenance
• Impenetrable to rodents and other undesired animals

Typical feeders are designed to distribute small, dry, consistently-shaped grains that can easily flow. However, gorilla feed consists of large irregular chunks of fruit and vegetables whose rough edges and wet surfaces cause jamming and sticking. In a traditional feeder, gorilla feed would cluster into immobile and inconsistent units of food and probably jam the feeder. In the ForageFeeder design, the feed is separated into individual servings, solving the jamming and portion control issues. The device is composed of the feed bucket, electronics housing, and associated wiring shown in Fig. 1. We discuss each in turn.

The feeder bucket consists of a 5-gallon (19 L) bucket, a metal funnel, an encoded motor, 3D-printed components, and laser-cut components. The feed bucket stores and separates feed into servings. In a feed cycle, the gate rotates and releases a serving of feed, which then falls from the feed bucket onto a toothed flywheel. When suspended 10 m off the ground, feed is thrown across a circular area with a radius of 10 m. Cycles are activated at random intervals on a timescale chosen by the user.

The electronics housing consists of laser-cut acrylic components, a motor, and associated electronics. Pieces are secured with nuts and bolts and waterproofed with silicon caulk.

The motors and Arduino Uno are powered by separate power banks. Power banks are reliable lithium (LiPo) batteries and do not have the danger of undercharging or overcharging the batteries. A full charge of the battery pack powering the motors can handle 24 feed cycles and can remain active for 24 h without charging or swapping the batteries out. Battery packs may conveniently be removed by opening the sliding panel at the back of the electronics box and unplugging their USB connections. In the next section, we present our detailed design choices.

### Feed Housing

The feed housing is a 5-gallon deer feed bucket with 19 cm-tall cylindrical dividers (12.7 cm x 19 cm acrylic panels) that separate the volume into eight wedges. Of the eight wedges of space, only seven wedges are filled with feed because one wedge acts as the initial “closed” position for the device. With each slot holding 1.5 L of feed, the seven wedges hold a total of 8 L.

**Fig. 2:**
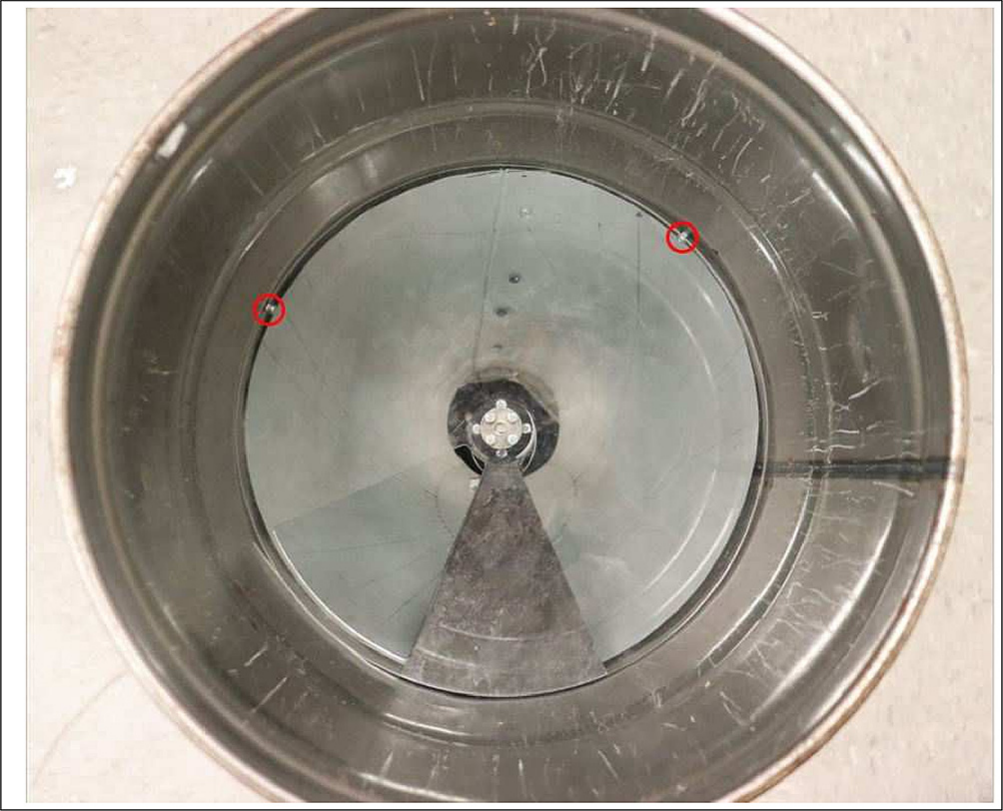
Additional tabs under panel gate for support.

The divider panels sit atop a circular acrylic panel with a wedge-shaped cutout that acts as a gate. While in the closed position, the cutout aligns with an empty wedge blocked off by the motor holder. In this position, there are no open spaces for the feed to fall through. To distribute feed, the panel rotates, shifting the cutout slot to a wedge filled with feed, which is subsequently released. Rotation is powered by an encoded DC motor held in place by a motor holder. A metal funnel is placed at the bottom of the bucket to help guide feed to the exit hole. To support the weight of heavier feed, M3 screws are screwed into the metal bucket right under the height of the panel gate (Fig. 2).

### Electronics Housing

The electronics housing hangs directly under the feed housing with four M6x300 fully threaded bolts. The housing is made of acrylic, silicon sealant, and cyanoacrylate. Inside the housing are the electronic components, interface components, and batteries. An Arduino Uno contains all the code to run the device. On top of the housing is a high-speed motor with a toothed flywheel that launches feed outwards during feeding.

Protruding on the front side of the housing is an LCD interface and controls for adjusting input settings. The manual controls include three buttons. As a safety precaution, an LED light flashes 10 min prior to all motors activating.

### Motor Specifications

Two motors generate the forces necessary to distribute food across a large area. The first motor, M1, is located directly below the feed and slowly spins the acrylic disk 45 degrees between each food distribution cycle. The hole in the disk lines up with one of the eight feeding compartments filled with food, allowing the food to fall down into the lower portions of the device. The motor has an appropriate accuracy and maximum torque in order to rotate the acrylic disk at the required speed and precision for successful deployment. Our motor had a torque of 300 kgf-cm, sufficient to spin the acrylic disk while supporting a full 3-kg load of feed. For the feed to be distributed properly, the disk should spin at least 16 rpm. Our chosen motor spins at 23 rpm, so we lowered the speed of rotation through pulse width modulation (PWM) from the Arduino Uno. In order to accurately spin the disk, an encoder is attached to the shaft of the motor. Every time the shaft makes one rotation, the encoder sends signals to the Arduino which counts the rotations. Using simple PID control, we spin the motor the needed 45 degrees.

The second motor, M2, spins the metal flywheel to launch the feed. We chose this motor so that it can give the flywheel enough torque to distribute feed up to 10 m from the device. This motor has a max rotational speed of 12,000 rpm and pulls at most 2.4 amps of current when in use by the feeder. PWM signals from the Arduino Uno are also used to control the speed of this motor. (See Table 1).

**Table 1:** Bill of Materials

**Designator**	**Component**	**Number**	**Unit Cost – USD**	**Total Cost – USD**	**Source of Materials**
Bucket	5 Gallon Bucket	1	$22.42	$22.42	MSCDirect
M1 Motor	23 RPM HD Premium Planetary Gear Motor w/Encoder	1	$59.99	$59.99	ServoCity
Motor Driver	Cytron 10A Dual Channel Bi-Directional DC Motor Driver	1	$25.80	$25.80	Amazon
Arduino Uno	ELEGOO UNO R3	1	$16.99	$16.99	Amazon
Arduino Shield	Custom Arduino Shield	1	$17.70	$17.70	JLCPCB
Portable Phone Charger	Anker Ultra Compact High Speed Voltage Boost Technology	1	$21.99	$21.99	Amazon
Rechargeable Battery	TalentCell Rechargeable 12 V 3000mAh Lithium ion Battery Pack	1	$26.99	$26.99	Amazon
M2 Motor	High Speed 12 V Motor	1	$18.88	$18.88	Amazon
Cord Protector	Cord Protector Wire Loom Tubing	1	$9.99	$9.99	Amazon
Zip Ties	Zip Ties	1	$6.99	$6.99	Amazon
Motor Coupler	6 M Motor Shaft Motor Coupler	1	$7.87	$7.87	Amazon
1/8” Acrylic Sheet	1/8” Cast Acrylic 12x24	5	$17.70	$88.50	McMaster
7/32” Acrylic Sheet	7/32” Cast Acrylic 12x24	3	$26.68	$80.04	McMaster
Silicone Caulk	Gorilla Glue Silicone Sealant Caulk	1	$9.84	$9.84	Amazon
Extension Adaptor	Extension Adaptor	1	$5.99	$5.99	Amazon
M3	M3 Set	1	$5.36	$5.36	Amazon
Long Bolt	1/4” x 6 inch Hex Bolt	4	$0.70	$2.80	Home Depot
1/4” Hex Nut	1/4” Nut	8	$0.09	$0.72	Home Depot
M5 Nut	M5 Nut	1	$0.94	$0.94	Home Depot
Washer	Washer Kit	1	$8.99	$8.99	Amazon
M5X25	M5X25 Bolt	1	$0.85	$0.85	Home Depot
M10X35	M10X35 Bolt	1	$1.77	$1.77	Home Depot
M10 Nut	M10 Nut	1	$0.72	$0.72	Home Depot
Funnel Sheet Metal	0.05” Thick, 24” x 24” Multipurpose Aluminum	1	$58.76	$58.76	McMaster-Carr
				**Total**	$**442.13**

Note: some items are only sold in bulk, and cost may vary. ” denotes one inch or 2.5 cm

### Electronics and Controls

The Arduino microcontroller is the brain of the device. A custom Arduino Shield printable circuit board (PCB) was designed using KiCad and printed using JLCPCB (Shenzhen JIALICHUANG Electronic Technology Development Co.,Ltd). A protoboard-based circuit may also act as a shield, which easily and compactly connects the Arduino to all the peripherals and motors. The Arduino Shield PCB is like a middle man that relays information between the microcontroller and the other devices. The Arduino Shield also mechanically binds different electronic modules with screws or male - female connections making the device more durable and the parts easy to replace. By sending the Gerber file to a manufacturer, the Arduino Shield PCB may be created and delivered in a timely manner and at an affordable price.

The Arduino Shield serves as a structural link between the Arduino and peripheral devices like the LCD display and buttons, preventing wires from becoming loose as ForageFeeder is moved. The male pins from the display fit tightly into the female connectors on the Arduino Shield. The female connects on the Arduino also fit into the male pins on the Arduino Shield. Using screws, we physically secured these peripherals to the Arduino Shield, which in turn was connected to the body of the device. Using screw terminals connects the Arduino to other devices securely. Other options like jumper cables going directly into the Arduino would not be as reliable.

We select a dual motor driver because it simplifies the wiring management along with making it more compact. The driver is a Cytron 10A dual channel bi-directional DC motor driver that can handle high peak current draws of 15 amp from the motor.

### Software Overview

The software handles everything from controlling the motors to timing the wait between feedings. The software is located in an Arduino Uno, a popular microcontroller that when powered starts executing the file in its Flash Memory. The executed code follows the format of calling a setup function when booted and then looping indefinitely in a loop function. However, a simple loop function would not work for our complex problem, so we use a state machine that segments the different actions the device takes. We describe the code segments in turn.

When the device is turned on and the Arduino is powered, the device goes into a configuration state. The configuration state takes inputs from three buttons and outputs information to the LCD display. In the case of the gorilla feeder, the number of feedings and hours active are displayed on separate lines. Two buttons are responsible for changing these variables. The third button changes the state from configuration mode to the setup state. During the setup state, the device stays idle for one hour to allow for the device to be secured in its intended location.

By pressing the third button, users can go back to the configuration state. After an hour, the state machine switches to the active wait state where the device waits 10 min before distributing feed. During this state, a red warning LED shines to users, after which the state machine switches to the active state where the food is distributed.

To distribute the feed, the Arduino sends out pulse width modulation signals (PWM) to the motor driver to drive the M2 motor attached to the toothed flywheel. Increasing the motor speed increases the distribution radius of the feed. A PWM signal also moves the slow spinning motor in order to drop the food in a wedge. Once the device goes through all its cycles, it then goes to the low power mode where it goes into deep sleep to preserve battery.

The main file that runs on the Arduino Uno is written in C++. Our code is written in a low-level language that is then compiled and uploaded to the Arduino Uno using the Arduino IDE. The serial rate is set at 9600 bits per second. We also use an library, the Liquid Crystal library. This library aids with the communication between the arduino uno and the LCD display.

To properly interpret button presses, a debounce function ensures an inconsistent button press doesn’t cause accidental double clicks. The debouncing function records the time a button is initially pressed, checks if the button remains pressed after a 100 ms delay, then waits for the button to be unpressed to execute the button’s command.

## Design files summary

**Table:** 

**Design filename**	**File type**	**Open source license**	**Location of the file**
Gorilla_Feeder_Main.ino	Arduino (.ino) File	GNU GPL v3	OSF
Funnel.7inHeight.SLDPRT	CAD file	GNU GPL v3	OSF
Motor_holder_v.4.SLDPRT	CAD file	GNU GPL v3	OSF
Bucket_v.3.SLDPRT	CAD file	GNU GPL v3	OSF
Full_v4.SLDASM	CAD file	GNU GPL v3	OSF
Schematic.kicad_pro	Kicad Project file	GNU GPL v3	OSF
Schematic.kicad_PCB	Kicad PCB file	GNU GPL v3	OSF
Schematic.kicad_sch	Kicad Schematic file	GNU GPL v3	OSF
Schematic.drl	Drill Data file	GNU GPL v3	OSF
Schematic-job.gbrjob	Gerber Job file	GNU GPL v3	OSF

• Gorilla_Feeder_Main.ino contains the code that runs on the Arduino and controls the inputs and outputs of the feeder.
• Funnel.7inHeight.SLDPRT is the 3D model for the funnel which is located at the bottom of bucket and focuses all the food so that it falls onto the flywheel.
• Motor_holder_v.4.SLDPRT is the 3D model for a component inside the bucket that connects the slow spinning motor to the bucket’s body.
• Bucket_v.3.SLDPRT is a 3D model for the bucket which holds the feed, the feed compartments, and the slow-spinning M1 motor.
• Full_v4.SLDASM is the 3D model for the entirety of the electronics box which contains the Arduino, PCB, user interface, motor driver, and batteries.
• Schematic.kicad_pro is the general project file for the entirety of the PCB that connects all the electronics. This file can be opened with the opensource software Kicad in order to modify the PCB design.
• Schematic.kicad_PCB is the file that contains the Kicad PCB design which can be opened in Kicad if the user wishes to change PCB routing or component placement.
• Schematic.kicad_sch is the schematic file that may be used to modify the components in the circuit schematic to change the PCB.
• Schematic.drl is the drill file that describes the mounting and via hole positions and sizes. This file is used in the manufacturing processes.
• Schematic-job.gbrjob is the Gerber job file used in the manufacturing process to etch the routes in the PCB.

## Build instructions

Prior to assembly, collect all materials, including cut pieces, 3D-printed pieces, nuts/bolts/washers, and adhesives/sealants. Thread the motor with encoder’s wires through the cord protector and cut to length (see Table 2).

1. Prepare the upper motor, M1, and motor holder (Fig. 3).
  A. Align the flat ends of the top of the motor, M1, with the flat sides of the motor holder. Ensure the wires face towards the motor holder. Thread all the wires through the center of the motor holder and encapsulate them between the motor and motor holder by pushing in the M1 motor into the motor holder.
  B. Screw in four M3x8 screws at the top of the motor holder to securely attach it to the motor.
    OPTIONAL STEP: Tighten a hose clamp around the base of the M1 motor and motor holder. This clamp is for extra security but is not necessary.
  C. Connect the end cap of the motor holder to the motor holder with cyanoacrylate. Seal the edge between the end cap and motor with silicone sealant or hot glue.
  D. Attach the motor adaptor to the motor shaft by aligning the set screw with the flat end of the motor shaft and tightening the set screw. The top of the motor shaft should be flush with the top platform of the motor adaptor.
    NOTE: By this point the M1 motor should be secure in the motor holder. Make sure that no wires are pinched, and all wires are threaded through the bottom of the motor holder. Make sure there is nothing that will prevent the motor shaft and adapter from rotating fully.

**Fig. 3:**
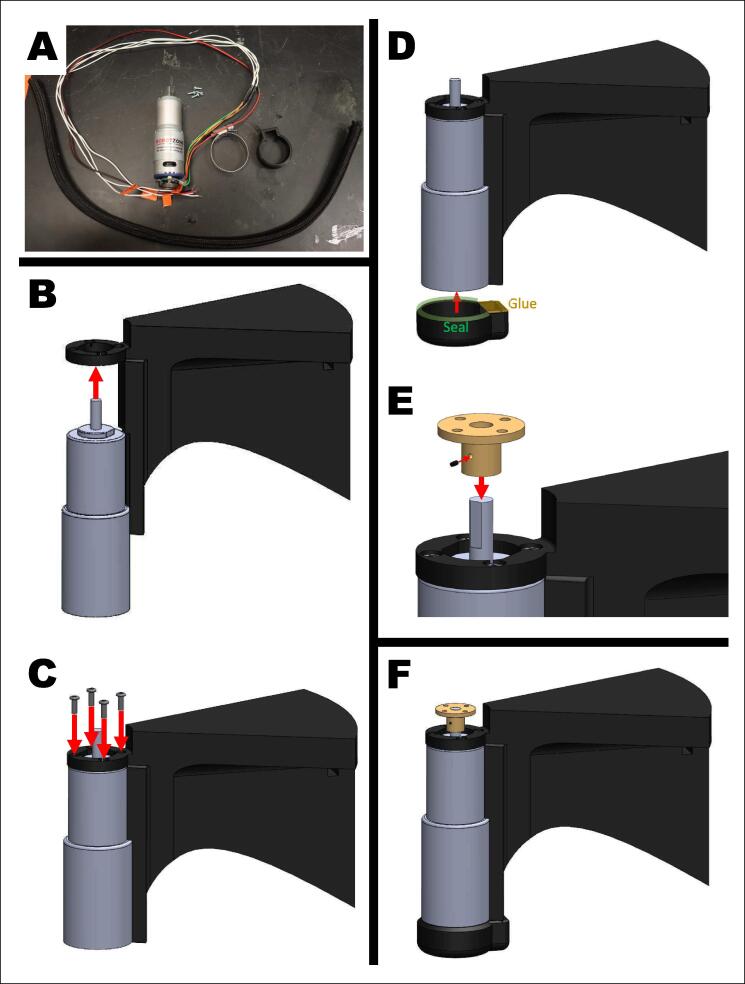
Building the upper motor system. A) Materials: motor, motor holder, end cap, hose clamp, four M3x8 screws, cord protector, and motor adaptor. B) Align the motor into the motor holder, thread wires through the motor holder, and push into place. C) Fasten the motor in place with four M3x8 screws. D) Seal the end cap of the motor holder to the motor holder at the highlighted locations. E) Place the motor adaptor on the motor shaft. Align the flat edge of the motor shaft with the set screw of the motor adaptor. Tighten the set screw. F) Completed motor system.
2. Attach the motor holder to the bucket (Fig. 4).
  A. Pair one M5 wave washer (or lock/spring washer) with one M5 hexnut. Drop them together into the cut out on the side of the motor holder. Use a small screwdriver to make sure that the washer and nut are snug inside the cut out. Also ensure that the holes align with the screw path. These pair of wave washers and hexnuts will act as the backing of the top screw attaching the motor holder to the bucket.
  B. Align the holes in the motor holder with the pre-drilled holes of the bucket. Screw to secure.
    NOTE: The top bolt and bottom bolt face opposite directions. The top M5 bolt’s hexnut is inside of the bucket while the bottom M10 bolt’s hexnut is outside of the bucket. Follow the washer, spring washer, and nut order in Fig. 4B.
    CHECK IN: Thread all wires through the wiring hole at the bottom of the bucket. This can be done at any time but is easiest now.
  C. Insert the four long screws into the bucket. The head of the bolts should be on the inside of the bucket. Secure the four long screws with one 1/4” hex nut each. The bottom of the long screws will be the attachment point of the electronics box.
  D. Place the funnel into the bucket. Angle the funnel into the bucket and under the motor holder as shown in Fig. 4C. The cut slit in the metal funnel will align with the underside of the M1 motor.
    NOTE: The wires should be under the funnel and through the underside of the bucket by this point.

**Fig. 4:**
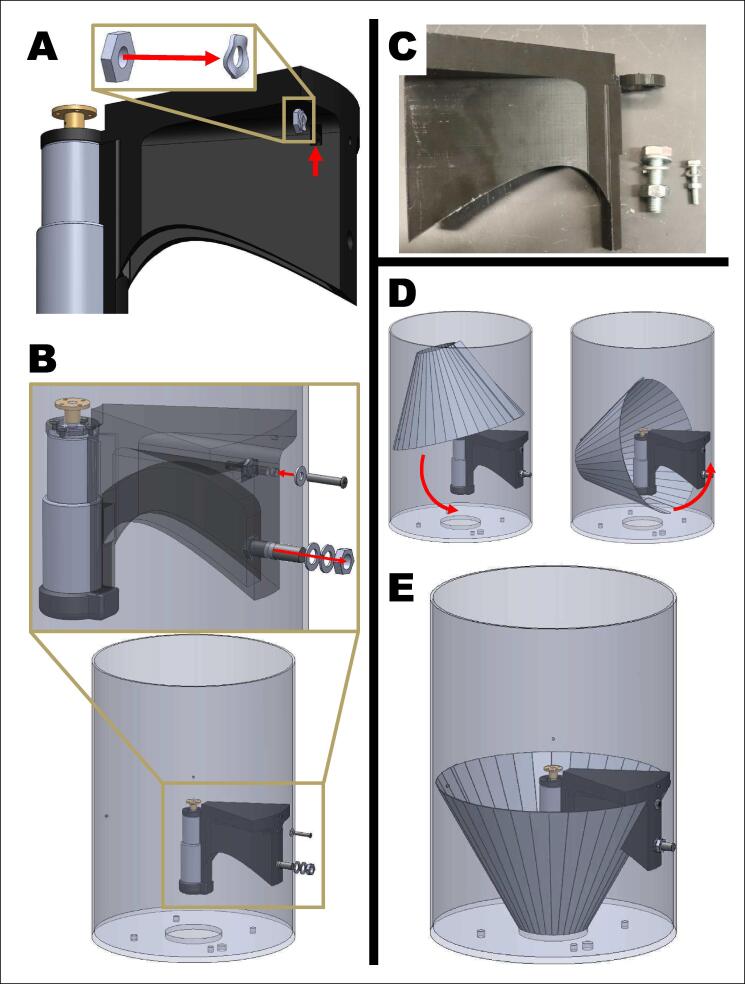
Attaching the upper motor system to the bucket. A) Pair one M5 wave washer and one M5 hexnut and drop inside hole of motor holder. B) Secure the motor holder to the bucket with M5 and M10 bolts in the directions shown. C) Materials: motor holder, M10 bolt, M10 spring lock, M10 washer, M10 nut, M5 bolt, M5 washer, and M5 nut. D) Insert funnel into place. E) Completed motor system inside bucket.
3. Wire all electronic controls according to the wiring setup (Fig. 5).

**Fig. 5:**
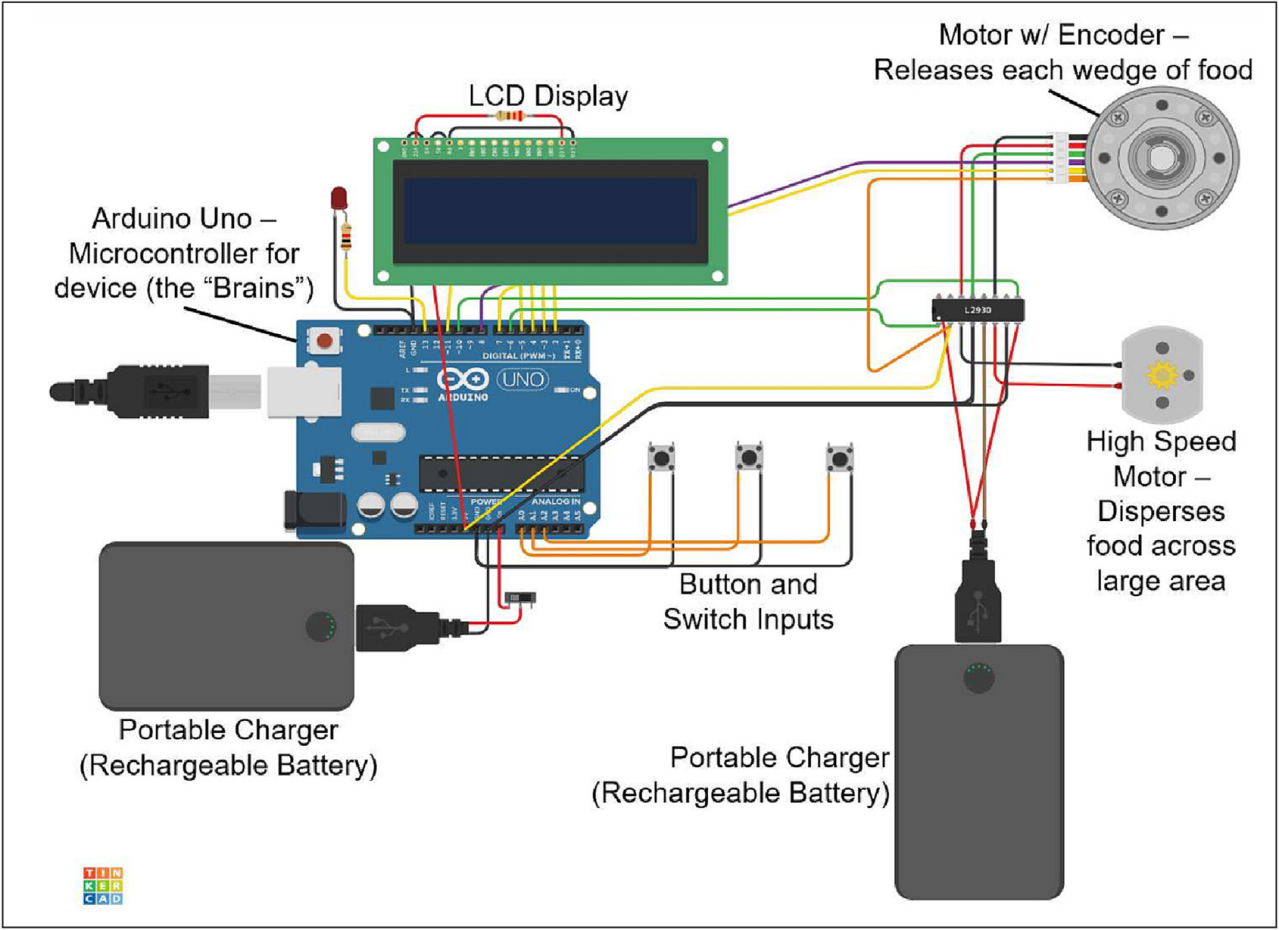
Overview of the main electronic components for ForageFeeder. Components can be categorized into the user interface (LCD display and buttons), processing units (Arduino and motor driver), actuators (motors), and electrical units (batteries and wires).
4. Prepare the electronics housing (Fig. 6).
  A. Build the outer waterproof acrylic housing by attaching the front, left, and right sides to the bottom of the housing. Use cyanoacrylate to secure each side and silicone caulk to seal each edge.
  B. Push zip ties through the attachment slots for the buttons.
  C. Install the LCD screen. Secure the screen in place with four M3x16 screws and four M3 nuts. Tighten the zip ties around the buttons to secure them in place.
    NOTE: DO NOT over tighten the screws and zip ties, this can cause hardware malfunction.
    NOTE: Add a small dot of hot glue to each screw and nut to prevent loosening.
  D. Hot glue the LCD into the LCD hole on the front panel.

**Fig. 6:**
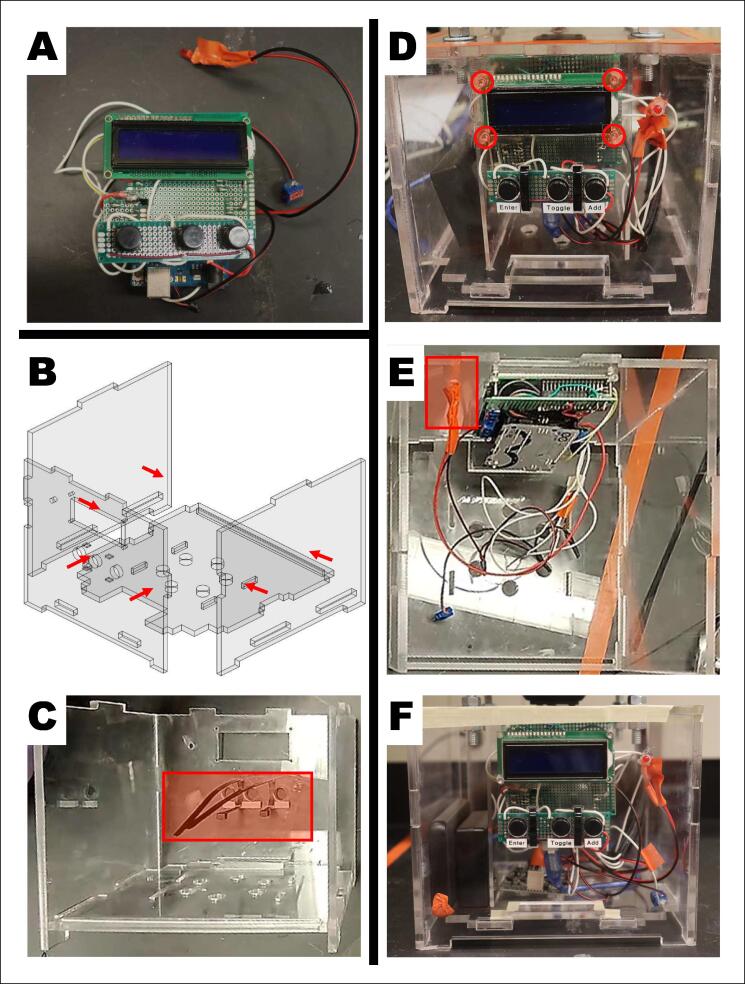
Assembly of the electronics housing and securing the electronic controls. A) Electronic components related to the user interface, processing units, and electrical units. B) Four of the electronic housing panels being fitted together. C) Secure the button panel to the electronic housing’s front panel with zip ties. D) Screw hole location where four M3 screws will secure the LCD display to the electronic housing’s front panel. E) Warning LED location on the electronics housing front panel. F) Completed electronics housing.
5. Seal the electronics housing (Fig. 7).
  A. Secure the fast motor, M2, to the top panel with two M3x10 bolts.
  B. Attach the toothed flywheel to the M2 motor shaft by aligning the set screw with the flat end of the motor shaft and tightening the set screw.
  C. Insert the door into the slot on the top electronics housing panel.
  D. Secure the top panel into place on the four long screws on the bottom of the bucket. Use the order of washers and nuts as seen in (Fig. 7). The top panel should sit at least 12 cm from the bottom of the bucket.
    NOTE: Add a small dot of cyanoacrylate to each nut and bolt to prevent loosening.
  E. Wire all wires according to the wiring guide and the electronics housing.
  F. Insert the inside panels in the electronics housing. This will help divide the electronics from the batteries.
  G. Attach the top panel (with bucket) onto the electronics housing. Make sure that the tube of wires is on the side with the door. The wires will go through the side of the door. Seal all edges of the top panel with silicon caulk. Extra tape can be used to secure the two together

**Fig. 7:**
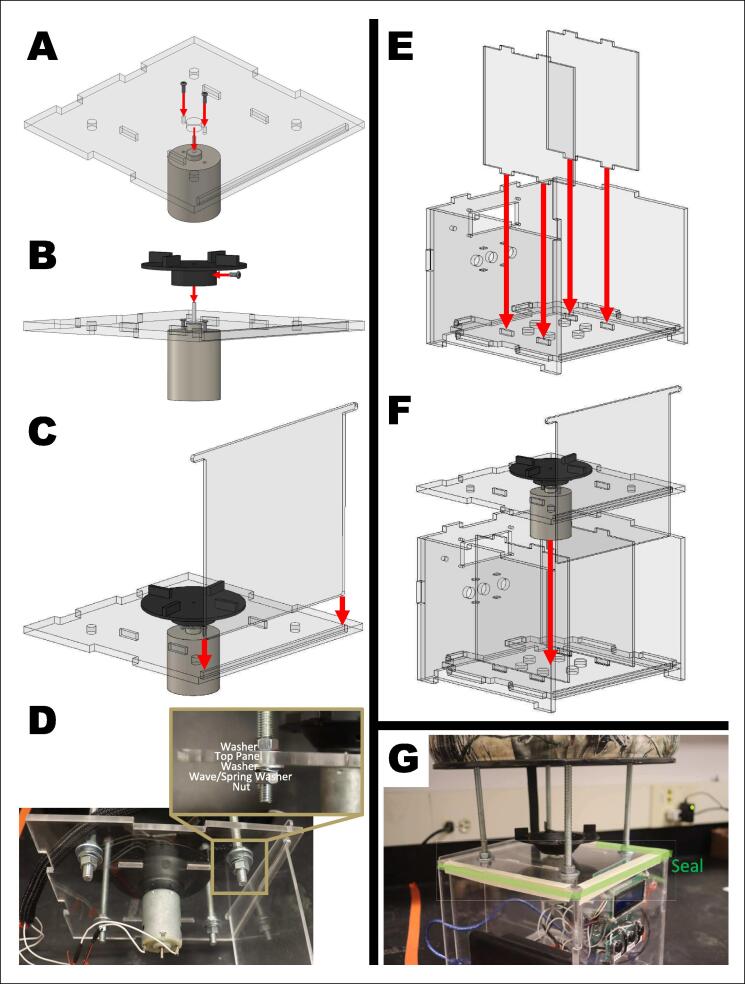
Sealing the electronics housing. A) Secure the fast motor to the top panel of the electronics housing. B) Secure the toothed flywheel onto the fast motor shaft. C) Insert the door into the top panel slot. D) Hardware order for securing top panel to long screws. E) Insert the internal dividers. F) Place top panel onto the electronic housing. G) Locations to seal with adhesive and sealant.
6. Set the divider system (Fig. 8).
  A. Set the slot cut panel onto the M1 motor system and align the four holes near the center of the panel. Insert one M3 screw into each hole. They do not need to be tightened, just placed flush against the panel.
  B. Place the borders and the center divider onto the slot-cut panel. The borders round the side of the bucket while the center divider sits in the center. The narrow end of the center divider is flush with the panel.
    NOTE: Ensure that each gap in the borders aligns with each gap in the center divider.
  C. Insert each acrylic divider into each gap of the divider system.
  D. Rotate the divider system so that one divided wedge aligns with the edges of the motor holder.
    NOTE: Do not rotate the slot cut panel. It is electronically calibrated during every start-up.

**Fig. 8:**
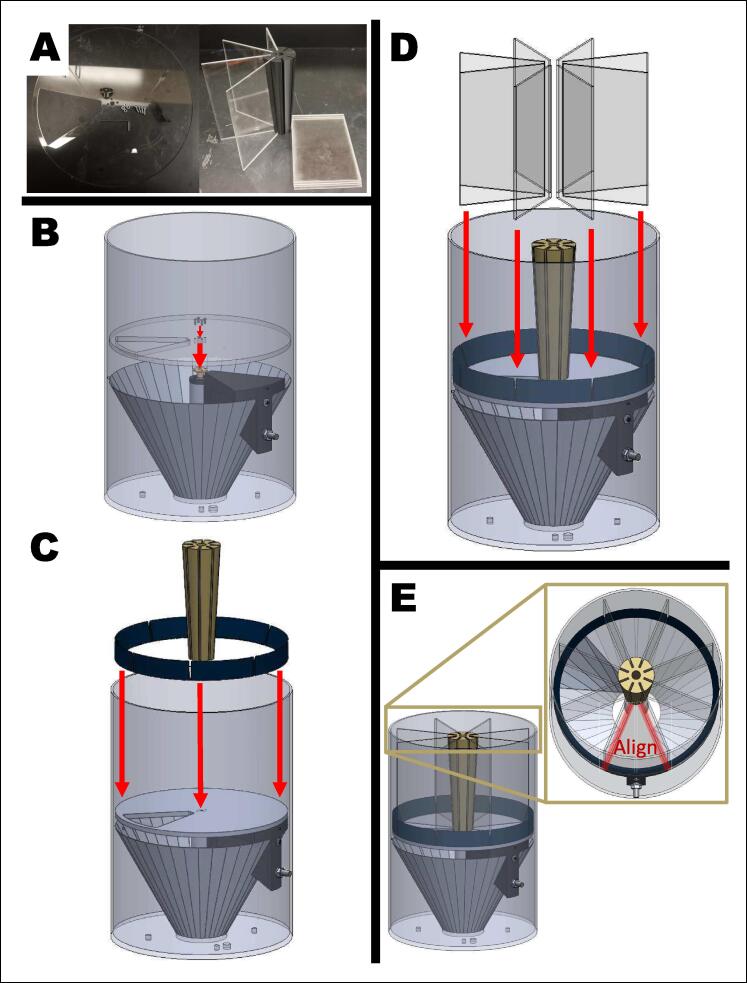
Completing and calibrating the device. A) Materials: slot cut panel, four M3 screws, borders, circular center divider, and eight acrylic dividers. B) Set the slot cut panel onto the motor system and align the four holes near the center of the panel. Insert one M3 screw into each hole and flush against the panel without tightening. C) Place the borders (blue) and the center divider (yellow) onto the slot-cut panel. The borders rim the bucket while the center divider sits in the center. The narrow end of the center divider is flush with the panel. NOTE: Ensure that each gap in the border aligns with each gap in the center divider. D) Insert each acrylic divider into each gap of the divider system. E) Rotate the divider system so that one divided wedge aligns with the edges of the motor holder. NOTE: Do not rotate the slot cut panel. It is electronically calibrated during every start-up.

**Table 2:** 3-D Printable Components

**Designator**	**Component**	**Number**	**Weight–g**	**Material type**
Motor Holder	MotorHolder_Body1.STL MotorHolder_Body1.STL	1	149 g	Tough PLA
Center Dispenser	Center_Dispenser_v.4.STL	1	178 g	Tough PLA
Borders	Body8.STL	4	27 g	Tough PLA

## Operation instructions

### Device Usage

**Fig. 9:**
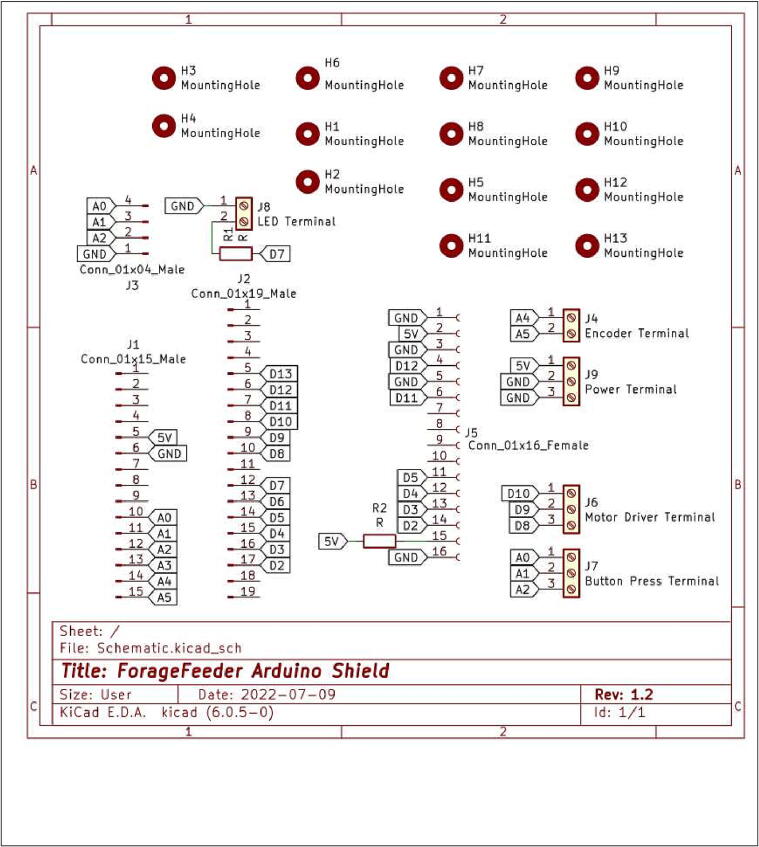
Circuit diagram for the Arduino Shield showing the various terminals and labelled trace ends that connect all the electronic components to one another.
2. Now, the display should display the calibration scene. During calibration, the rightmost two buttons under the display may be used to calibrate the acrylic disk underneath the dividers so that the opening is lined up onto the black plastic armature. By pressing the middle button (Toggle), the acrylic disk spins counterclockwise. Pressing the rightmost button (Add) makes the acrylic disk spin clockwise.
3. The feed can now be filled into the slots on the top of the device. The slot that faces the triangular opening in the acrylic disk should be left empty.
4. Pressing the leftmost button (Enter button) ends the calibration and advances the device to the next stage.
5. Now, the display should be on and showing the Food Cycles and Total Hours options.
  (a) The line with asterisks (*) indicates the currently selected option. Pressing the middle button (Toggle), to change the selection.
  (b) The rightmost button (Add button) increases the value of the currently selected option
  (c) The left most button (Enter button) will activate the device and advance to the next stage. Display items include:
  • Food Cycles: The number of clusters of food to be distributed (maximum is seven)
  • Total Hours: The total hours the device is feeding. Maximum is 12 h, excluding the one hour of idle time given to set up the device.
6. After pressing the leftmost button to activate the device, a 60-min countdown begins on the display. During these 60 min, pressing the leftmost button returns the user to the Food Cycle and Total Hours setup screen.
7. After the 60 min wait time, the device goes through the feeding time cycles.
8. When the device is going through feeding cycles, a red LED on the face of the electronics box will light up to indicate that food will be distributed within 10 min. Do not touch the device during this time.
9. Once the feeding is done, the batteries should be unplugged or turned off to disable the device. The batteries should be recharged or replaced if they are low.

**NOTE:** *Before each deployment, check for screws loosened by motor vibration.*

1. Turn on and plug in two battery packs. Charge if power indicators are low.
  • Important: Turn on and slowly double click Anker PowerBank’s power button to enter low power mode. One of the LEDs on the button should turn from blue to green.
  • Make sure the other BatteryPack is switched on (LEDs should be green) (See Fig. 9)

### How Feeding Time are Distributed

To calculate the time for each feeding, divide the total hours by the number of food cycles. For example, two total hours and seven food cycles yields 2/ 7 ≈ 17 min per feeding.

### Device Care and Cleaning

All components in the bucket are removable for cleaning. Do not disassemble the upper motor system or detach from the bucket once built. Instead, remove all other components and rinse the inside of the bucket. Wipe the outside of the Electronics Housing to clean. The toothed flywheel can be removed and reattached for cleaning.

## Validation and characterization

Currently the capabilities of the device include:

• dispersing cubic-inch (2.5 cm length) food chunks
• dispersing in a 10-m radius
• operable for approximately 13 h at highest load

We endeavored to build an automated feeder that can be easily reproduced and modified. ForageFeeder is open-source, meaning that all our work is documented and publicly available to be used or modified. In addition, collaboration between users is also possible through the OSF (open source framework) page where all the software code is stored. We kept the device safe and robust by keeping all the sensitive electronics in a water resistant portion of the device that remains accessible for maintenance and cleaning. For the power, we used battery packs with built in safeties and circuitry to control charging and shutting off the lithium cells to reduce the risk of fires if a short circuit or excessive current draw occurs. The batteries powering the electronics will last approximately 38 and a half hours as the battery has a capacity of 10000 mAh and the electronics draw an average of 260 mA (42 mA from Arduino, 200 mA from LCD display, 18 mA from extraneous components like resistors). At the highest power consuming feeder settings (7 feeding cycles an hour), the battery powering the motors will last 13.9 h as the battery has a capacity of 3000 mAh and the motors and motor driver draw around 216 mA (average 116 mA from the motors and 100 mA from the drivers).

The main electronic component controlling the whole device is an Arduino Uno which is a user-friendly microcontroller popular in education and hobbyist electronics. The electronics are also easy to source and assemble. Our OSF page includes a Gerber file for a PCB that connects all the electronic components. This Gerber file can be used by third parties like JLCPCB to manufacture the electronic circuits. This custom circuit reduces manufacturing as the connections use male or female pin connectors or screw terminals. To modify the device, for example, a speaker that buzzes before food is delivered or LORA module that allows for remote feeding can be added. The PCB design would need to be edited, or the new components hand-soldered in. Libraries are easy to install from the Arduino Uno IDE and provide time-saving features and documentation for free.

In August 2022, our team worked with zoo keepers to walk through a build of the device from start to finish, a process that took four hours. The device was satisfactorily constructed, with adequate rotation and timing between the two motors, leading to successful feeding. This feeder has been at gorilla habitat at Zoo Atlanta for over four months. At randomly designated intervals, it spreads 7 L of sweet potatoes across the habitat each day (Supplemental Video 2). The feeder holds a maximum of 10L of cut fruit or vegetables. The 7L of sweet potato provides 5600 kcal of food. Since a single gorilla’s diet is 950–8000 kcal a day, our feeder provides a useful portion of food to an entire gorilla troop in a captive setting, and reduces the manual feeding by zoo staff. The results of this long-term feeding experiment will be published in a follow-up work.

## Conclusion

We designed and built ForageFeeder, an open-source feeder for use in animal research and conservation. Unlike other automated feeders, our feeder dispenses large food chunks that are consumed by gorillas. This device takes only a few hours to build, is approximately $400, and is easy to maintain and operate. We hope that our device will provide animal enrichment as well as access and inspiration for future open source conservation technology tools.

## CRediT authorship contribution statement

**Nima Jadali:** Software, Data curation, Writing - original draft. **Margaret J. Zhang:** Conceptualization, Methodology, Writing - original draft. **Andrew K. Schulz:** Writing - review & editing. **Josh Meyerchick:** Visualization, Investigation. **David L. Hu:** Writing - review & editing.

## Declaration of Competing Interest

The authors declare that they have no known competing financial interests or personal relationships that could have appeared to influence the work reported in this paper.
